# Leaf-Root Trait Coordination Among Dominant Herbaceous Species Across Three Marsh Habitats in Northeast China

**DOI:** 10.3390/plants15172661

**Published:** 2026-08-30

**Authors:** Qiuyu Meng, Shilin Liu, Jiping Liu, Yanhui Chen

**Affiliations:** College of Geographic Science and Tourism, Jilin Normal University, 1301 Haifeng Street, Siping 136000, China; mqy@mails.jlnu.edu.cn (Q.M.); 1848509451@mails.jlnu.edu.cn (S.L.); cyh@jlnu.edu.cn (Y.C.)

**Keywords:** plant functional traits, different types of wetlands, adaptation strategies, trade-off relationships, Northeast China

## Abstract

Plant functional traits are key indicators of plant responses to environmental conditions and resource-allocation trade-offs, thereby providing insights into adaptive mechanisms across habitats. Wetlands are one of the major habitats worldwide, yet organ-level plant life adaptive strategies in different wetland types remain insufficiently understood. This study compared three representative marsh systems located in different regions of Northeast China. Forty-five plant community plots were established, and 10 leaf traits, 13 root traits, and 8 soil environmental variables were systematically measured for dominant herbaceous species. We examined above- and belowground trait trade-offs using Pearson correlation analysis and assessed their responses to environmental gradients using redundancy analysis (RDA). The results showed that trait variability differed markedly among marsh types. Inland salt marshes exhibited the greatest variation in leaf and root morphology, while morphological traits generally varied more strongly than ecophysiological traits. In inland salt marshes, root ecophysiological traits were closely associated with leaf morphology. Forested marshes showed multi-trait trade-offs and nutrient regulation, whereas freshwater herbaceous marshes displayed strong leaf-root trait correlations, indicating flexible inter-organ nutrient allocation. Soil environmental properties explained different proportions of functional trait variation across habitats, with the highest explanatory power in forested marshes (42.85%), followed by freshwater herbaceous marshes (34.56%) and inland salt marshes (34.41%). Soil carbon-to-nitrogen ratio significantly affected plant growth in all three habitats, although the other environmental drivers were habitat-specific. These findings reveal distinct community-level patterns of leaf–root trait variation and coordination among the sampled dominant herbaceous species across the three marsh habitats, providing empirical evidence for understanding wetland plant adaptation mechanisms in Northeast China.

## 1. Introduction

Plant functional traits link environmental change, plant responses, and ecosystem processes, and are widely used to reveal adaptive mechanisms across habitats [1]. In wetlands, these traits indicate habitat evolution and ecosystem functions [2]. Trait studies are often framed by the leaf economics spectrum (LES) and root economics spectrum (RES), which describe trade-offs between resource acquisition and conservation in leaves and between nutrient uptake and tissue maintenance in roots [3,4,5]. Relationships between wetland-specific traits and these spectra vary with environmental stress, reflecting multiple trade-offs between resource acquisition and stress tolerance [6]. Under persistent water saturation, wetland plants tend to adopt resource-acquisitive strategies [7]. However, whether environmental stress weakens or strengthens aboveground–belowground trait coordination remains contested. Some studies report that nutrient limitation or heterogeneous soil conditions decouple leaf and root traits because aboveground and belowground organs respond to different limiting resources [8,9], whereas others indicate that strong environmental filtering, such as persistent saline stress, may strengthen coordination between particular dimensions of leaf and root traits [10].

The generalized economic spectrum extends this framework to the whole-plant level, emphasizing coordinated adaptation among organs under environmental filtering [11]. Although research has progressed beyond single gradients or organs, coordinated aboveground–belowground adaptation across multiple wetland types at regional scales remains insufficiently understood [12]. In particular, the direction and threshold of shifts among coupling, trade-offs, and decoupling along wetland stress gradients have not been resolved.

Wetland habitats are particularly important in China; Northeast China alone contains 7.5 × 10^4^ km^2^ of wetlands, accounting for 14.1% of China’s wetland area and 48.3% of its marshes [13]. In China, studies have advanced understanding of wetland plant traits. Xu Le et al. explained floating mat formation in raft-sedge wetlands [14]; Sang Lian et al. showed that leaf carbon and root phosphorus jointly regulate soil carbon and nitrogen [15]; and An Subang et al. revealed reed trait responses to disturbance [16]. However, most studies focus on single stresses or organs. Given big environmental differences among wetland types, resource allocation trade-offs likely vary [17], yet coordinated leaf–root adaptation in Northeast China remains poorly studied.

There are still three knowledge gaps at present: whether the morphological and elemental proportion characteristics show consistent variation patterns under different levels of marsh stress; whether the coordination between leaves and roots will systematically shift from coupling to decoupling as the stress intensity increases; and whether there is a common soil driving factor that regulates different organs through specific habitat pathways. Solving these gaps is necessary to link the theory of low-nutrient-level ecosystems and high-nutrient-level ecosystems with the overall adaptability of plants in heterogeneous wetland habitats.

Northeast China hosts extensive inland marshes, including freshwater herbaceous marshes in the Sanjiang Plain, salt marshes in the western Songnen Plain, and forest marshes in the Greater and Lesser Xing’an Mountains, forming key temperate wetland systems and ecological barriers [18]. Based on preliminary literature review and field surveys, representative areas of these three marsh systems were selected for comparative study. This study examines dominant herbaceous plants across these habitats by measuring leaf and root traits and associated soil conditions. We addressed three questions: (1) how do leaf and root trait variability differ among wetland habitats; (2) how does aboveground–belowground trait coordination change across habitats; and (3) which soil factors drive habitat-specific adaptive strategies? Correspondingly, we hypothesized that: (H1) morphological traits were expected to exhibit pronounced variability, particularly in inland salt marshes, whereas carbon stoichiometric traits were expected to remain relatively stable; (H2) under high saline–alkaline stress, leaf–root coordination was expected to show a less consistent pattern, while cross-organ coordination was expected to differ among the three marsh systems; and (H3) soil carbon-to-nitrogen ratio was expected to show recurrent associations with plant trait variation across the three marsh systems, with habitat-specific relationships among traits and soil conditions.

## 2. Results

### 2.1. Trade-Off Relationships Among Functional Traits of Herbaceous Plants in Different Types of Wetlands

#### 2.1.1. Correlation Analysis of Plant Traits in Inland Salt Marshes

**Figure 1:**
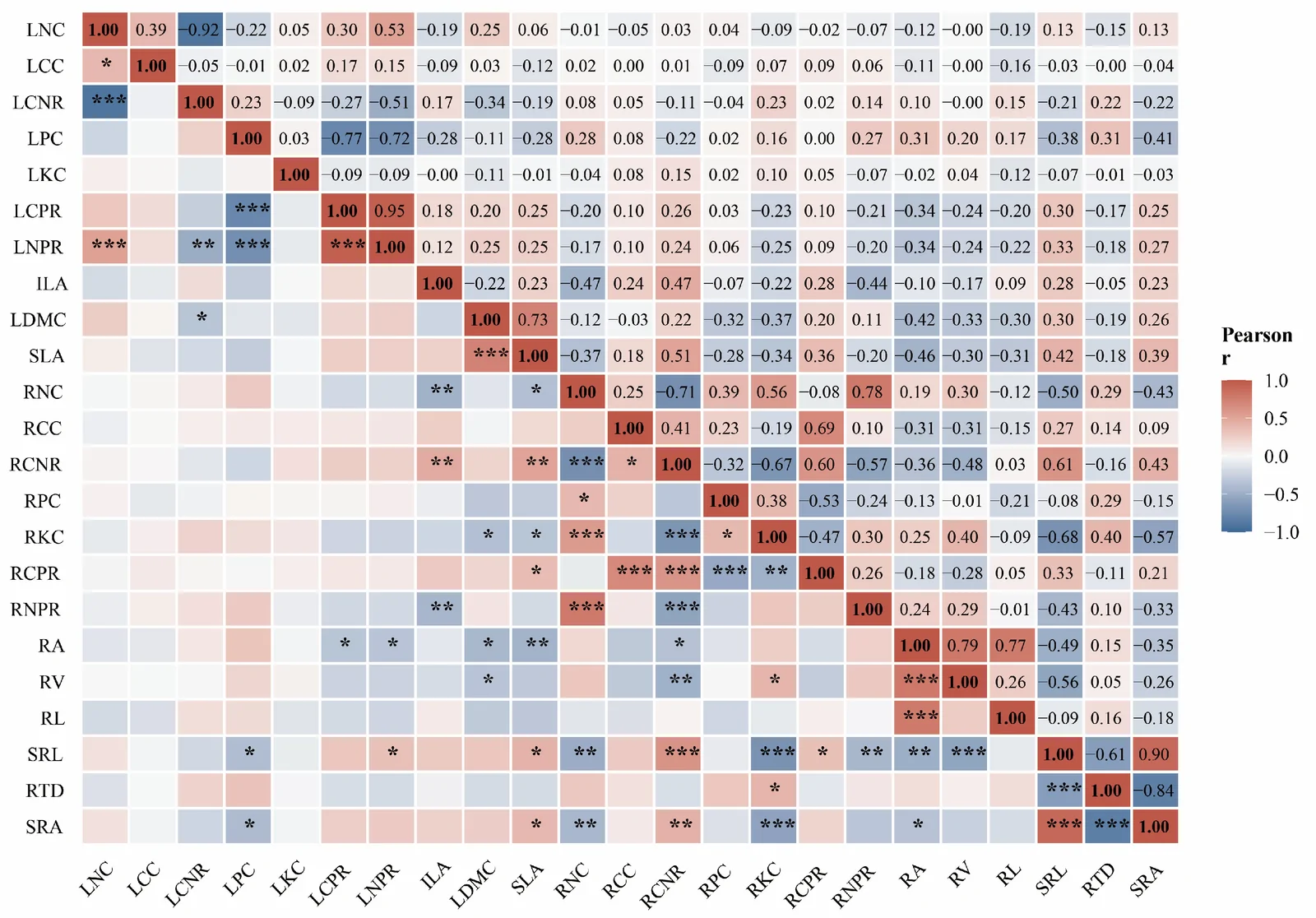
Correlation Analysis of Leaf and Root Traits in Inland Salt Marsh Plants. Note: ILA, leaf area; SLA, specific leaf area; LDMC, leaf dry matter content; LCC, leaf carbon; LNC, leaf nitrogen; LPC, leaf phosphorus; LKC, leaf potassium; C:N, C:P, N:P, carbon-to-nitrogen, carbon-to-phosphorus, and -nitrogen-to-phosphorus ratios in leaves. RL, root length; RV, root volume; RA, root surface area; SRL, specific root length; RTD, root tissue density; SRA, specific root area; RCC, root carbon; RNC, root nitrogen; RPC, root phosphorus; RKC, root potassium. Statistical significance is indicated by asterisks, where *, **, and *** correspond to *p* < 0.05, *p* < 0.01, and *p* < 0.001, respectively.

Significant correlations between leaf and root traits in plants of the inland salt marshes within the Momoge Nature Reserve were detected (Figure 1). Among leaf traits, specific leaf area shows a significant positive correlation with leaf dry matter content; leaf nitrogen content is positively correlated with leaf carbon content and the leaf carbon-to-phosphorus ratio, while it is negatively correlated with the leaf carbon-to-nitrogen ratio. Regarding root traits, specific root area is positively correlated with specific root length and the root carbon-to-nitrogen ratio, while it is negatively correlated with root nitrogen and potassium content; root volume, however, is positively correlated with root nitrogen and potassium content. Also, correlation analysis between leaf and root traits revealed that root potassium content, root surface area, and root nitrogen content were negatively correlated with specific leaf area, whereas specific leaf area was positively correlated with specific root area, specific root length, and the root carbon-to-phosphorus ratio. This suggests the existence of synergistic and compensatory mechanisms between the aboveground and belowground parts of the plant in terms of resource acquisition and allocation. Because the Pearson correlations were calculated from plot-level observations pooled across dominant species, they represent community-level covariation rather than within-species relationships. Accordingly, the absence of significant correlations between most root morphological traits and other leaf traits (*p* > 0.05) is interpreted as community-level trait decoupling; it does not demonstrate independent evolution of organs, because species turnover among plots may also contribute to the observed pattern.

#### 2.1.2. Correlation Analysis of Plant Traits in Forest-Swamp Wetlands

**Figure 2:**
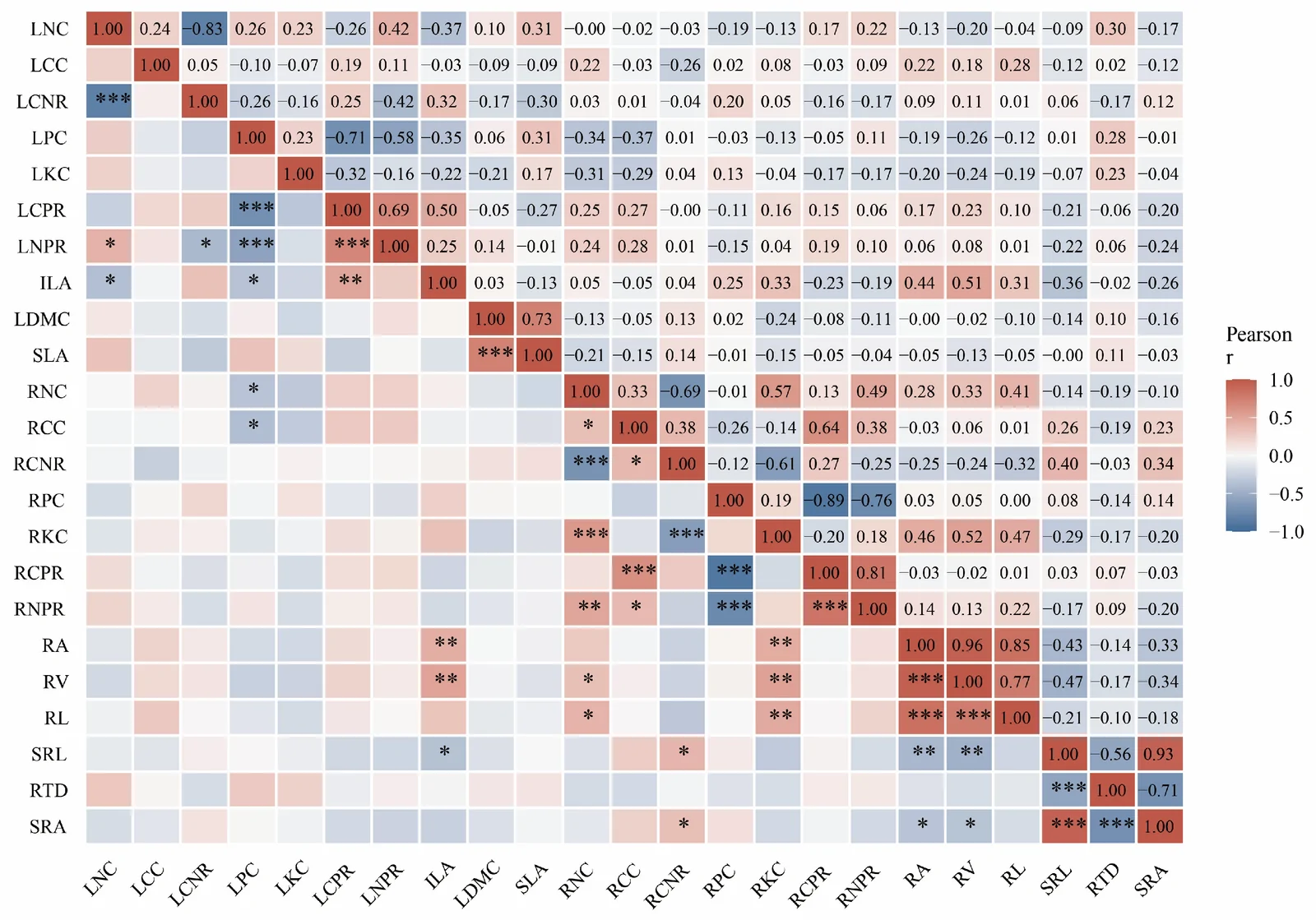
Correlation Analysis of Leaf and Root Traits in Forest Swamp Plants. Note: ILA, leaf area; SLA, specific leaf area; LDMC, leaf dry matter; LCC, leaf C; LNC, leaf N; LPC, leaf P; LKC, leaf K; C:N, C:P, N:P, leaf carbon-to-nitrogen, -phosphorus, and nitrogen-to-phosphorus ratios. RL, root length; RV, root volume; RA, root surface area; SRL, specific root length; RTD, root tissue density; SRA, specific root area; RCC, root C; RNC, root N; RPC, root P; RKC, root K; C:N, C:P, N:P, root carbon-to-nitrogen, carbon-to-phosphorus, and nitrogen-to-phosphorus ratios. Red, significant positive correlation; blue, significant negative correlation (*p* = 0.05). Numbers indicate correlation coefficients. Statistical significance is indicated by asterisks, where *, **, and *** correspond to *p* < 0.05, *p* < 0.01, and *p* < 0.001, respectively.

In the Youhao Marsh forested wetland, correlations between leaf and root traits of plants were also detected (Figure 2). Here, specific leaf area was positively correlated with leaf dry matter content and leaf nitrogen content, while leaf area was negatively correlated with leaf nitrogen content and leaf phosphorus content. Leaf phosphorus content was negatively correlated with the leaf carbon-to-phosphorus ratio and the leaf nitrogen-to-phosphorus ratio. Regarding root traits, specific root area was negatively correlated with root surface area, root volume, and root tissue density, and positively correlated with specific root length and the root carbon-to-nitrogen ratio. Root tissue density was highly significantly negatively correlated with specific root length, while root length was positively correlated with root potassium content, root surface area, and root volume. Correlation analysis between leaf and root traits showed that root volume, root surface area, and root potassium content were positively correlated with leaf area, while leaf phosphorus content and leaf potassium content were negatively correlated with root carbon content and root nitrogen content, reflecting a highly coupled relationship between the aboveground and belowground parts of the plant in terms of nutrient allocation. However, most root morphological traits showed no significant correlation with other leaf traits (*p* > 0.05).

#### 2.1.3. Correlation Analysis of Plant Traits in Freshwater Herbaceous Marshes

**Figure 3:**
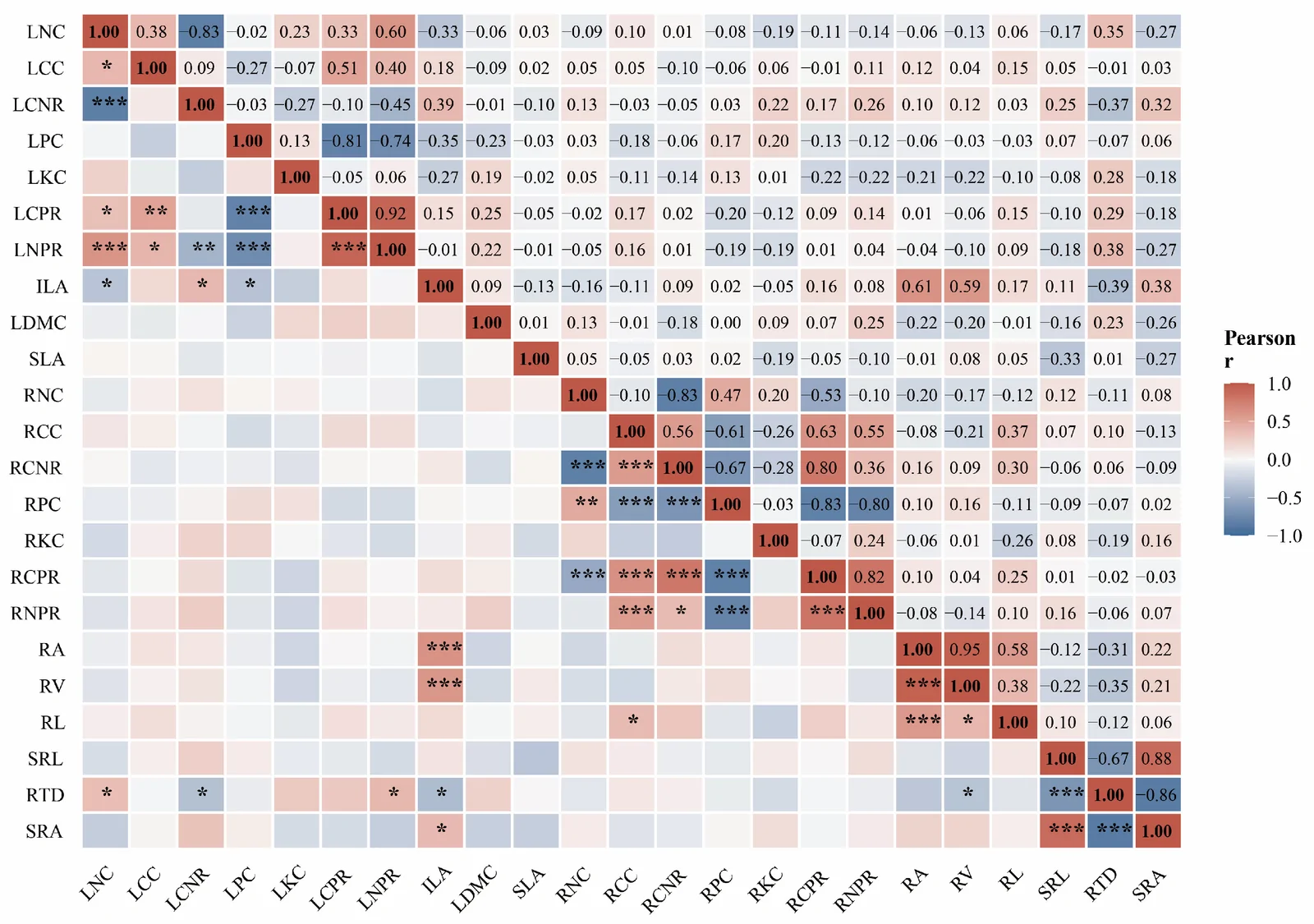
Freshwater Herbaceous Marsh Plant Leaf and Root Trait Correlation Analysis. Note: ILA, leaf area; SLA, specific leaf area; LDMC, leaf dry matter; LCC, leaf C; LNC, leaf N; LPC, leaf P; LKC, leaf K; C:N, C:P, N:P, leaf ratios. RL, root length; RV, root volume; RA, root surface area; SRL, specific root length; RTD, root tissue density; SRA, specific root area; RCC, root C; RNC, root N; RPC, root P; RKC, root K; C:N, C:P, N:P, root ratios. Red, significant positive correlation; blue, significant negative correlation (*p* = 0.05). Numbers indicate correlation coefficients. Statistical significance is indicated by asterisks, where *, **, and *** correspond to *p* < 0.05, *p* < 0.01, and *p* < 0.001, respectively.

In freshwater wetlands of the Sanjiang Nature Reserve watershed, the following correlations were detected among the tested traits (Figure 3): leaf area was negatively correlated with leaf nitrogen and phosphorus content; leaf nitrogen content was positively correlated with leaf carbon content; and the leaf carbon-to-phosphorus ratio was positively correlated with the leaf nitrogen-to-phosphorus ratio. Regarding root traits, specific root area was highly significantly positively correlated with specific root length and highly significantly negatively correlated with root tissue density; root length was positively correlated with root carbon content, root surface area, and root volume; root carbon content was highly significantly negatively correlated with root phosphorus content, and root nitrogen content was also highly significantly negatively correlated with root phosphorus content. Specific root length was negatively correlated with specific leaf area, while leaf area was positively correlated with specific root area, root volume, and root surface area, and negatively correlated with root tissue density. No significant correlation was observed between root ecophysiological traits and leaf functional traits (*p* > 0.05).

### 2.2. Analysis of Differences in Functional Traits of Herbaceous Plants in Different Types of Marshes

#### 2.2.1. Analysis of Environmental Characteristics and Differences Among Different Types of Marshes

**Figure 4:**
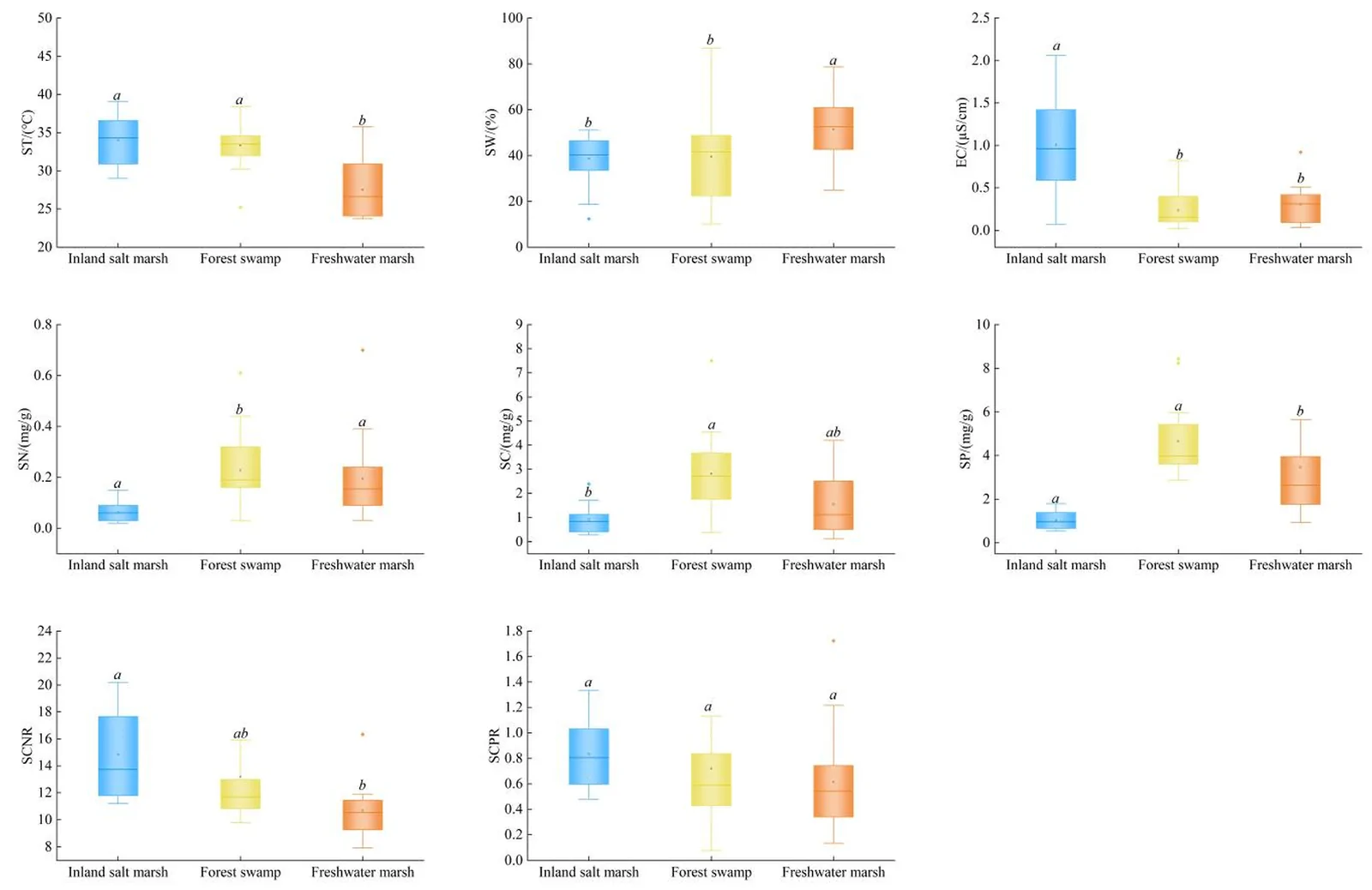
Comparison of the differences in environmental factors of various types of swamp wetland soils. Note: SW—soil moisture; ST—soil temperature; EC—soil electrical conductivity; SN—total soil nitrogen; SP—total soil phosphorus; SC—total soil organic carbon; SCPR—soil carbon-to-phosphorus ratio; SCNR—soil carbon-to-nitrogen ratio. In the box plot, the dots outside the upper and lower whiskers represent the observations that are beyond the range of 1.5 times the interquartile range (IQR). Different lowercase letters in the figure indicate statistically significant differences between groups at the *p* < 0.05 level (multiple comparisons were performed using the Tukey HSD test).

Wetland soil properties varied across the regions (Figure 4). The CV values for soil properties in inland salt marshes in the western part of the Songnen Plain range from 9.00% to 63.71%, while those in forested marshes in the Lesser Khingan Range range from 9.13% to 91.32%, whereas the CV values for soil characteristics in freshwater herbaceous marshes in the Sanjiang Plain range from 14.00% to 75.38%.

Analysis of variance (ANOVA) results indicate that soil temperature (ST) was significantly higher in the Songnen Plain and Lesser Khingan Range regions than in the Sanjiang Plain region (*p* < 0.05). Soil moisture (SW) was significantly higher in the Sanjiang Plain region than in the other two regions (*p* < 0.05), and soil temperature (ST) data in the Lesser Khingan Range region exhibited greater variability. The highest soil electrical conductivity (EC) values were observed in the western part of the Songnen Plain, significantly higher than in the other two regions (*p* < 0.05). Soil total nitrogen (SN), soil total phosphorus (SP), and soil total organic carbon (SC) were all significantly higher in the Lesser Khingan Range region than in the western Songnen Plain region and slightly higher than in the Sanjiang Plain region (*p* < 0.05). The soil carbon-to-phosphorus ratio (SCPR) and soil carbon-to-nitrogen ratio (SCNR) were significantly higher in the western Songnen Plain than in the Sanjiang Plain, and slightly higher than in the Lesser Khingan Range region (*p* < 0.05). Overall, soil environmental factors exhibited considerable variability, while soil nutrient status followed the order: Lesser Khingan Range region > Sanjiang Plain > western Songnen Plain.

#### 2.2.2. Comparative Analysis of Leaf Functional Traits in Different Types of Marshes

Significant differences were observed in the functional traits and stoichiometric characteristics of plant leaves across different types of marshes. Specifically, the CV values for leaf functional traits in inland salt marshes in the western part of the Songnen Plain ranged from 15.48% to 170.32%, while those in forest marshes in the Lesser Khingan Mountains ranged from 14.10% to 97.43%, while the CV values for leaf functional traits in freshwater herbaceous marshes in the Sanjiang Plain range from 13.52% to 82.60%.

**Figure 5:**
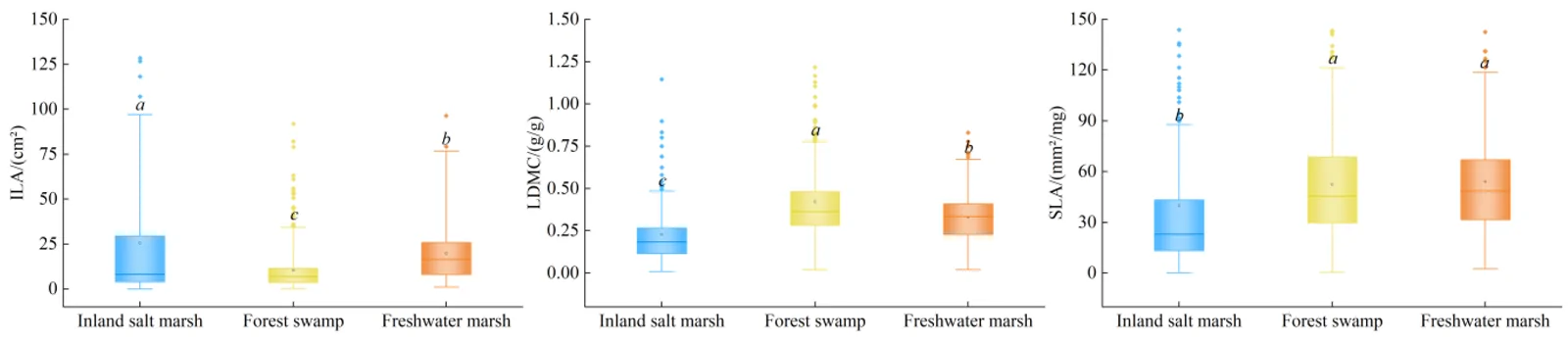
Differences in Leaf Morphological Traits of Different Types of Marshes. Note: ILA = leaf area; SLA = specific leaf area; LDMC = leaf dry matter content. In the box plot, the dots outside the upper and lower whiskers represent the observations that are beyond the range of 1.5 times the interquartile range (IQR). Different lowercase letters in the figure indicate statistically significant differences between groups at the *p* < 0.05 level (multiple comparisons were performed using the Tukey HSD test).

Leaf morphological traits varied significantly among the three wetland types (Figure 5). Herbaceous plants in the western Songnen Plain and the Sanjiang Plain exhibit better leaf conditions, with higher leaf area (ILA), slightly higher than that in the Lesser Khingan Range region. In the Lesser Khingan Mountains region, specific leaf area (SLA) and leaf dry matter content (LDMC) were slightly higher than those in the Sanjiang Plain region (*p* < 0.05), while the Sanjiang Plain region showed slightly higher values than the western part of the Songnen Plain.

**Figure 6:**
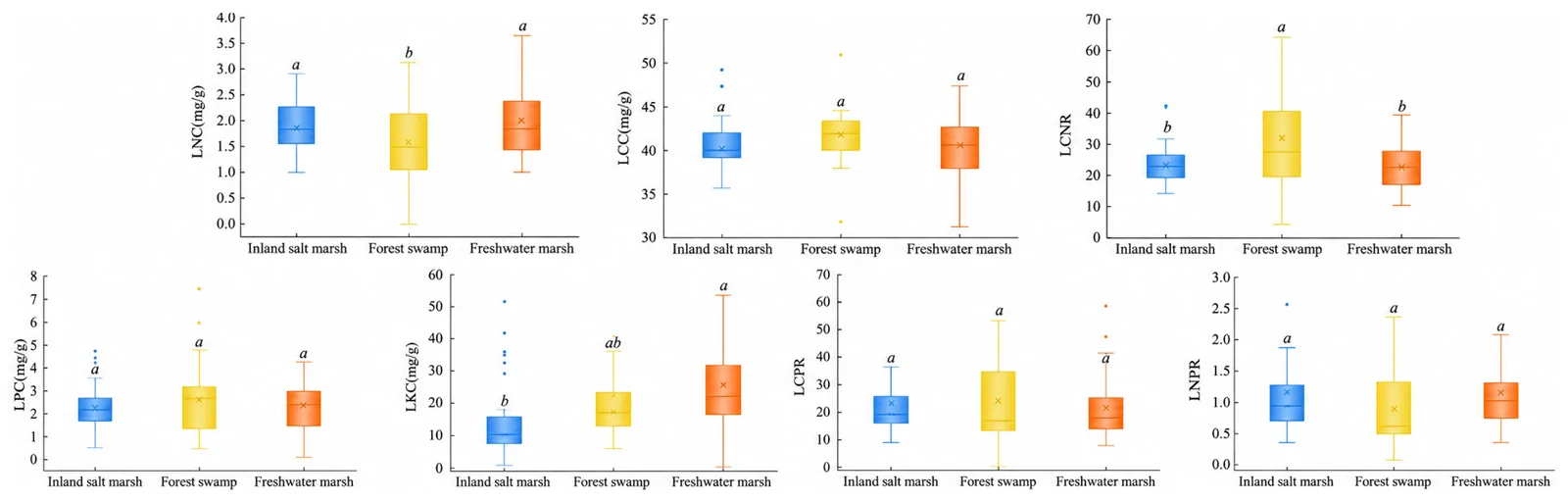
Differences in the stoichiometric characteristics of leaf blades in different types of swamp wetlands. Note: LCC—leaf carbon content; LNC—leaf total nitrogen content; LKC—leaf total potassium content; LPC—leaf total phosphorus content; LNPR—leaf nitrogen-to-phosphorus ratio; LCPR—leaf carbon-to-phosphorus ratio; LCNR—leaf carbon-to-nitrogen ratio. In the box plot, the dots outside the upper and lower whiskers represent the observations that are beyond the range of 1.5 times the interquartile range (IQR). Different lowercase letters in the figure indicate statistically significant differences between groups at the *p* < 0.05 level (multiple comparisons were performed using the Tukey HSD test).

The chemical composition of the leaves (Figure 6) indicates that the total carbon content (LCC), total phosphorus content (LPC), carbon-to-nitrogen ratio (LCNR), and carbon-to-phosphorus ratio (LCPR) of the leaves in the Lesser Khingan Range region were significantly different from those of the other two regions (*p* < 0.05). The total nitrogen content (LNC) in leaves was slightly higher in the other two regions than in the Lesser Khingan Range region (*p* < 0.05), but overall there was no significant difference. The total potassium content (LKC) in leaves was higher in the Sanjiang Plain region than in the Lesser Khingan Range region (*p* < 0.05), while the Lesser Khingan Range region was slightly higher than the western Songnen Plain region (*p* < 0.05). There were no significant differences in the leaf nitrogen-to-phosphorus ratio (LNPR) among the three regions (*p* > 0.05). Among the three different types of marshes, the leaves of herbaceous plants in the Lesser Khingan Mountains region exhibited higher carbon content, phosphorus content, carbon-to-nitrogen ratio, and carbon-to-phosphorus ratio. This indicates that the leaf tissue structure is relatively well-developed and the physiological condition is good; however, growth is prone to being inhibited by nitrogen content. In contrast, herbaceous plants in the western Songnen Plain region maintain growth in a relatively nutrient-poor environment by increasing internal nitrogen content. Leaves in the Sanjiang Plain exhibited higher potassium content, a functional trait consistent with the region’s abundant water supply, which facilitates potassium absorption and transport in plants.

#### 2.2.3. Comparative Analysis of Root Functional Traits in Different Types of Marshes

Significant differences in root functional traits and stoichiometric characteristics were observed across different types of marshes. Specifically, the CV values for root functional traits in inland salt marshes in the western part of the Songnen Plain ranged from 3.00% to 113.60%, the CV values for root functional traits in forest marshes in the Lesser Khingan Range range from 19.83% to 65.00%, and the CV values for root functional traits in freshwater herbaceous marshes in the Sanjiang Plain range from 14.31% to 91.69%.

**Figure 7:**
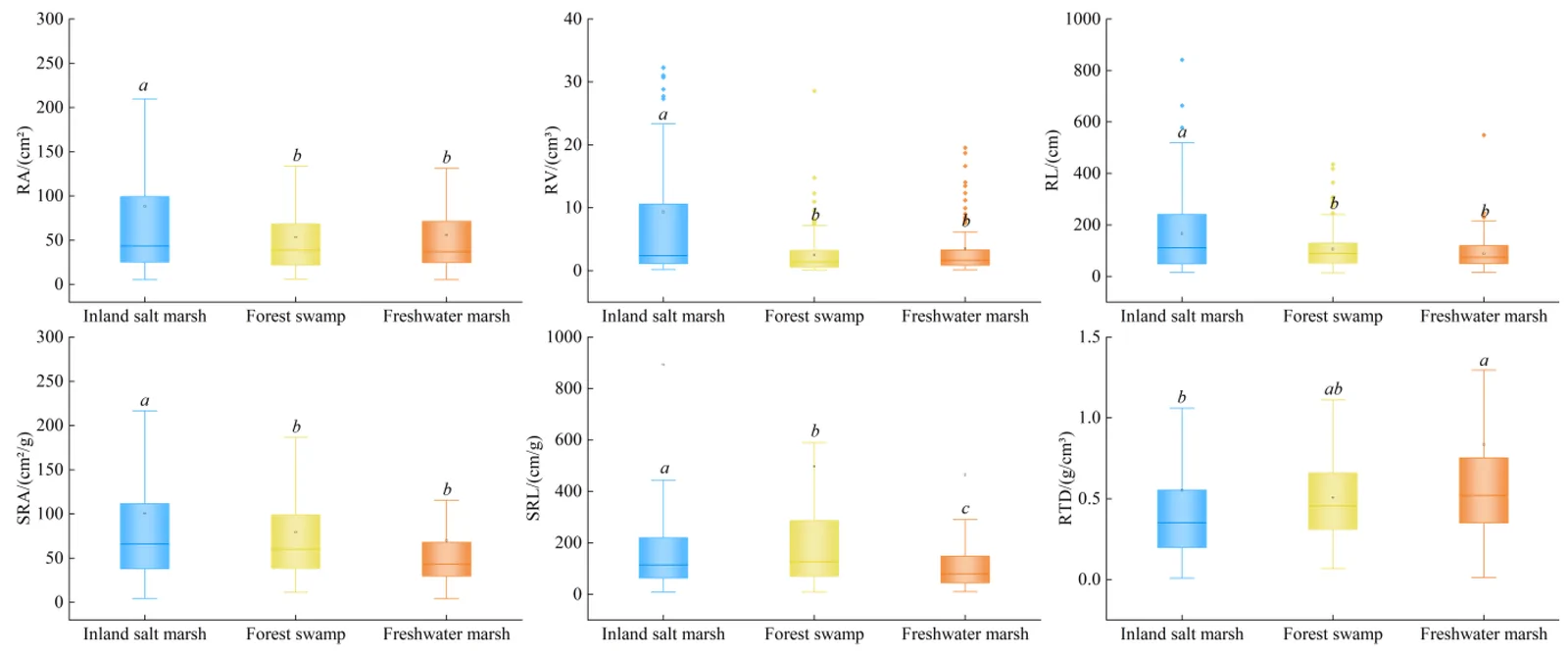
Differences in Root Morphological Characteristics of Different Types of Marshes. Note: RL—root length; RV—root volume; RA—root surface area; SRL—specific root length; RTD—root tissue density; SRA—specific root area. In the box plot, the dots outside the upper and lower whiskers represent the observations that are beyond the range of 1.5 times the interquartile range (IQR). Different lowercase letters in the figure indicate statistically significant differences between groups at the *p* < 0.05 level (multiple comparisons were performed using the Tukey HSD test).

Plant root morphological traits differed significantly among marsh types (Figure 7): specific root area (SRA), root volume (RV), root surface area (RA), and root length (RL) were slightly higher in the western Songnen Plain than in the other two regions (*p* < 0.05). Specific root length (SRL) was significantly higher in the Lesser Khingan Range region than in the other two regions (*p* < 0.05). The Sanjiang Plain and Lesser Khingan Range regions exhibited slightly higher root tissue density (RTD) than the Songnen Plain region (*p* < 0.05). In inland salt marshes, herbaceous plants have gradually developed robust, short, and well-developed root systems to adapt to high-salinity and alkaline environments. In contrast, the soil pH in freshwater herbaceous marshes and forest marshes is typically close to neutral, with relatively mild chemical properties. This environment is conducive to the normal growth of root cells and the formation of cell walls; root cells are able to divide and differentiate according to normal physiological rhythms, thereby forming tightly arranged, high-density tissues.

**Figure 8:**
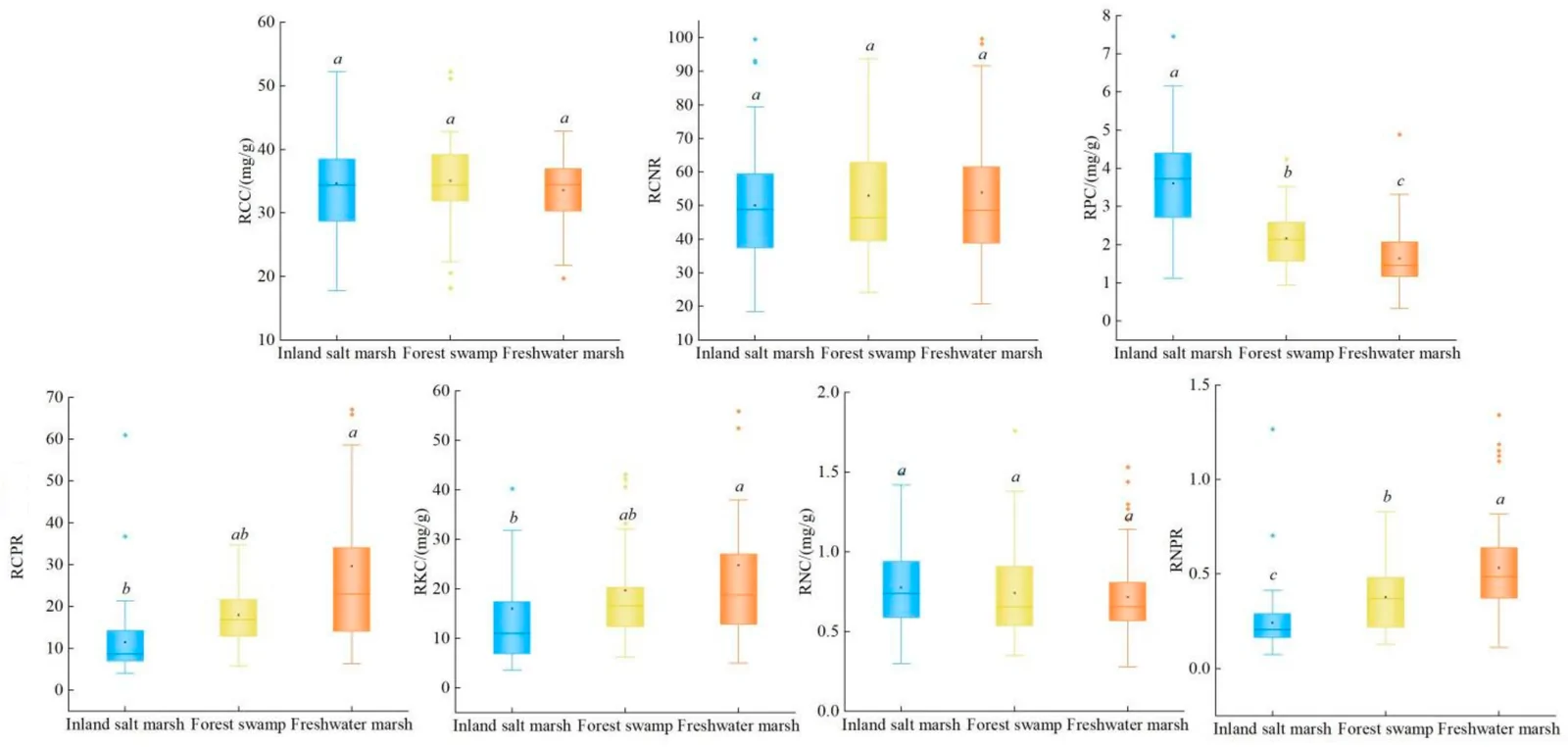
Differences in the stoichiometric characteristics of root systems in different types of swamp wetlands. Note: RCC—Total carbon content in roots; RNC—Total nitrogen content in roots; RKC—Total potassium content in roots; RPC—Total phosphorus content in roots; RNPR—Root nitrogen-to-phosphorus ratio; RCPR—Root carbon-to-phosphorus ratio; RCNR—Root carbon-to-nitrogen ratio. In the box plot, the dots outside the upper and lower whiskers represent the observations that are beyond the range of 1.5 times the interquartile range (IQR). Different lowercase letters in the figure indicate statistically significant differences between groups at the *p* < 0.05 level (multiple comparisons were performed using the Tukey HSD test).

Root stoichiometric traits showed relatively little variation among the three wetland types (Figure 8): there were no significant differences in root nitrogen content (RNC), root carbon content (RCC), or root carbon-to-nitrogen ratio (RCNR) (*p* > 0.05). Root phosphorus content (RPC) was significantly higher in the western region of the Songnen Plain than in the other two regions (*p* < 0.05). Regarding root potassium content, root nitrogen-to-phosphorus ratio, and root carbon-to-phosphorus ratio, the Sanjiang Plain region showed slightly higher values than the Lesser Khingan Range region (*p* < 0.05), while the Lesser Khingan Range region, in turn, showed slightly higher values than the western part of the Songnen Plain (*p* < 0.05).

### 2.3. Adaptive Strategies of Wetland Plants to Environmental Variations Based on Functional Traits

An RDA of plant functional traits and soil environmental factors in different types of marshes revealed the degree to which each soil factor explains the variation in each trait indicator.

#### 2.3.1. RDA Ranking of Plant Functional Traits in Inland Salt Marshes

**Figure 9:**
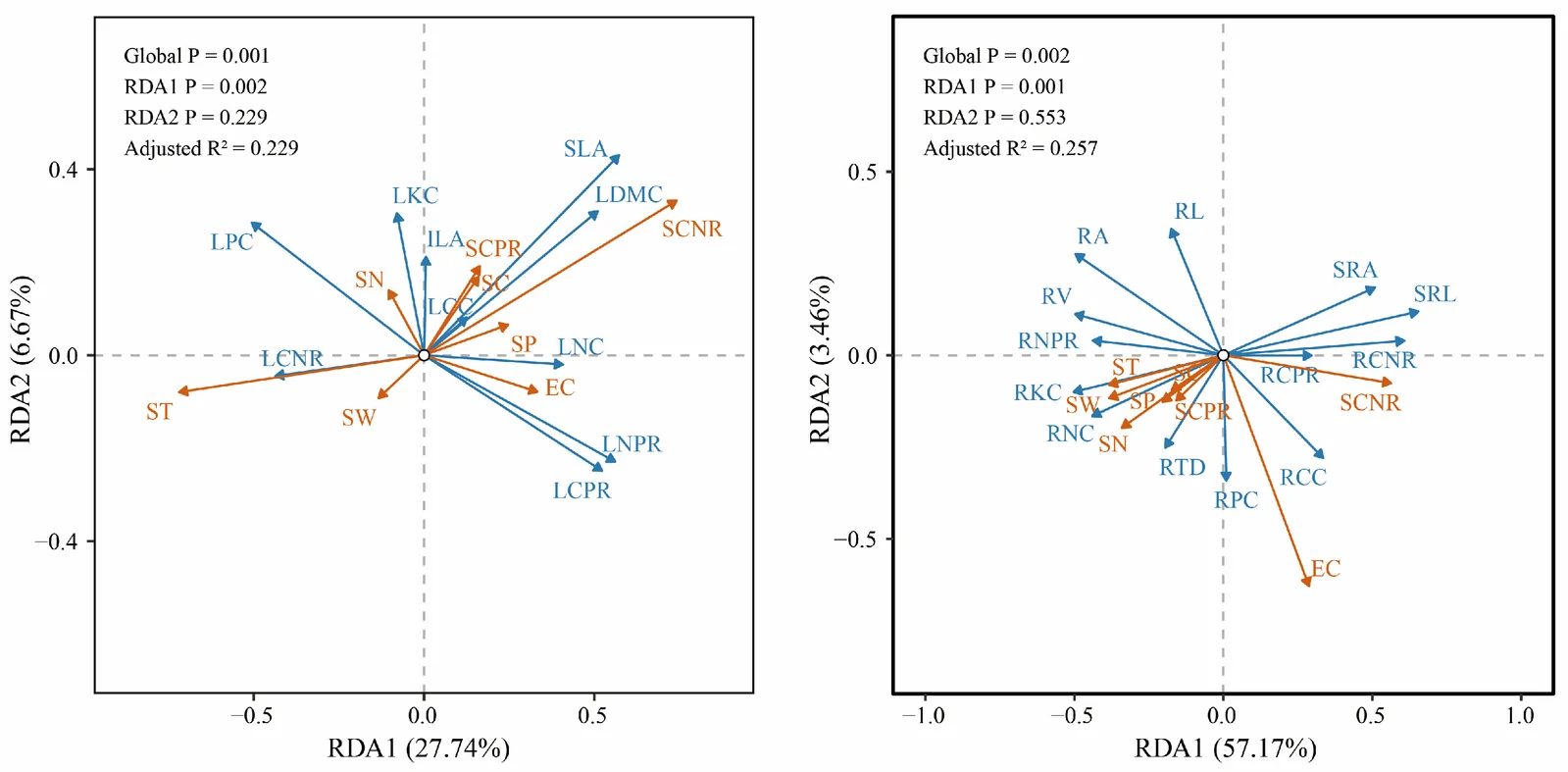
Ranking the Contributions of Plant Functional Traits in Inland Salt Marshes Based on RDA.

The RDA of leaf traits in inland salt marshes (left panel of Figure 9) shows that Axis 1 and Axis 2 explain 27.74% and 6.67% of the variance, respectively, with a cumulative explanatory power of 34.41%. The RDA of root traits (right panel of Figure 9) shows that Axis 1 explains 57.17% of the variance and Axis 2 explains 3.46%, with a cumulative explanatory power of 60.63%. This indicates that soil environmental factors explain a substantially higher proportion of variation in root traits than in leaf traits in this marsh system. Among these, soil temperature (ST) was significantly positively correlated with the leaf carbon-to-nitrogen ratio (LCNR); that is, as ST increased, LCNR tended to rise, reflecting the influence of temperature changes on the carbon–nitrogen metabolic balance in leaves. Soil temperature (ST) was positively correlated with root length (RL), suggesting that rising temperatures may promote root elongation; conversely, it was negatively correlated with root tissue density (RTD), implying that higher temperatures may reduce root tissue density and affect root architecture. Electrical conductivity (EC) was negatively correlated with multiple leaf traits, including total leaf carbon content (LCC) and leaf area (ILA), suggesting that factors such as soil salinity, as reflected by changes in EC, may inhibit leaf carbon accumulation and area expansion; EC was also negatively correlated with root carbon-to-nitrogen ratio (RCNR), indicating that increased EC may suppress RCNR, meaning that increased soil salinity may affect the carbon-to-nitrogen metabolic balance in the root system. The weak correlation between soil moisture content and leaf morphology suggests that moisture has some influence on leaf morphology but is not the dominant factor; however, soil moisture content (SW) is positively correlated with root potassium content (RKC), indicating that increased soil moisture content promotes potassium uptake and accumulation by the root system.

#### 2.3.2. RDA Ranking of Functional Traits of Plants in Forested Marshes

**Figure 10:**
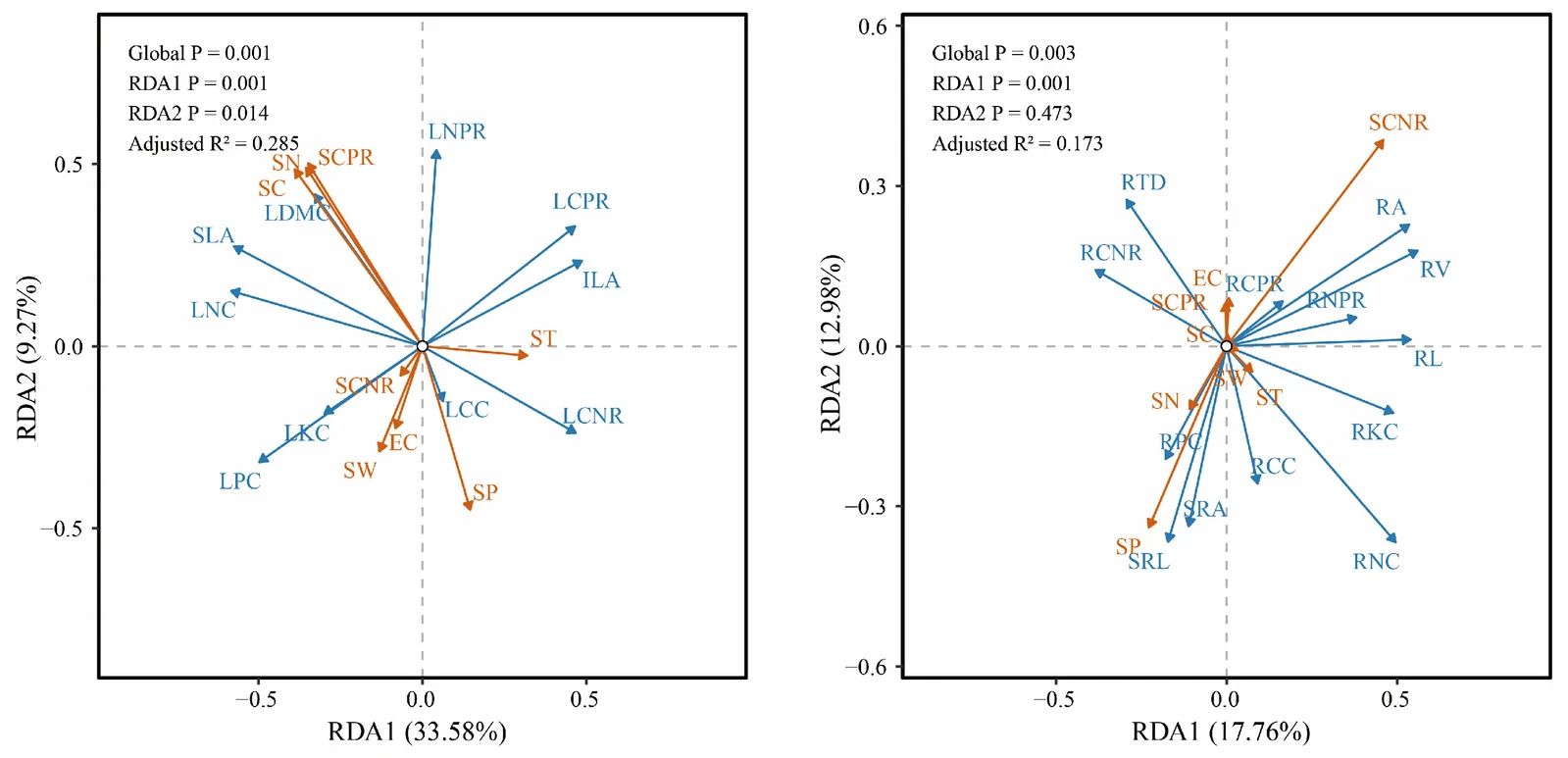
RDA Ranking of Plant Functional Traits in Forests, Swamps, and Wetlands.

The RDA results for plant leaves in forested marshes (left panel of Figure 10) show that Axis 1 explains 33.58% of the variance, Axis 2 explains 9.27%, with a cumulative explanatory power of 42.85%. The RDA of root traits in forested marshes (right panel of Figure 10) shows that Axis 1 explains 17.76% of the variance, Axis 2 explains 12.98%, with a cumulative explanatory power of 30.74%. This indicates that soil environmental factors and physicochemical properties influence leaf and root traits differently. Different soil factors influence various leaf and root traits in different directions and to varying degrees, reflecting the specificity of the plant’s aboveground and belowground responses to the soil environment. Notably, the leaf carbon-to-phosphorus ratio (LCPR) is negatively correlated with soil temperature (ST), implying that as soil temperature increases, the leaf carbon-to-phosphorus ratio may decrease; this suggests that temperature affects the plant’s processes of carbon and phosphorus uptake, metabolism, and allocation. Root tissue density (RTD) is negatively correlated with soil water content (SW); when soil water content is high, root tissue density decreases. This may be because sufficient water eliminates the need for the root system to develop an overly dense structure to access water, thereby leading to a decrease in tissue density. Complex relationships exist between multiple root traits and soil factors. Root surface area (RA) and root volume (RV) exhibit varying degrees of correlation with multiple soil physicochemical properties, indicating that root morphological characteristics are influenced by a combination of soil factors. Soil carbon, nitrogen, and phosphorus content, as well as water content and electrical conductivity, all play a role in root growth and morphological development.

#### 2.3.3. RDA Ranking of Functional Traits of Freshwater Herbaceous Marshes Plants

**Figure 11:**
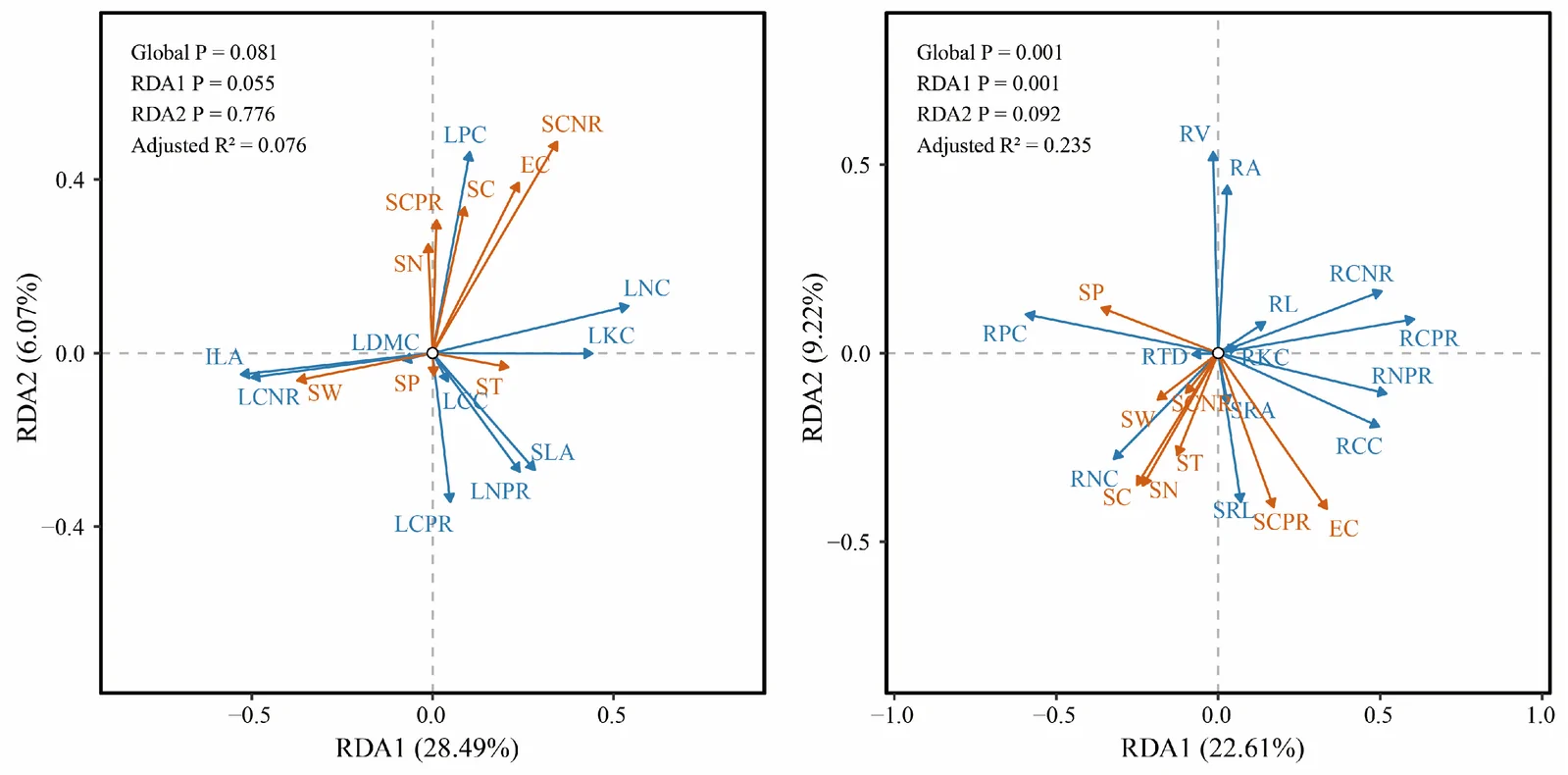
Ranking the Contributions of Plant Functional Traits in Freshwater Herbaceous Marshes Using RDA.

The RDA results for the leaves of freshwater herbaceous marsh plants (left panel of Figure 11) show that Axis 1 explains 28.49% of the variance, Axis 2 explains 6.07%, with a cumulative explanatory power of 34.56%. The RDA results for plant root traits (right panel of Figure 11) show that Axis 1 explains 22.61% of the variance, Axis 2 explains 9.22%, with a cumulative explanatory power of 31.83%. This suggests that variation in leaf traits may be influenced by relatively concentrated factors, whereas variation in root traits may result from the combined effects of a wider range of factors. Among these, the leaf carbon-to-nitrogen ratio (LCNR) was significantly positively correlated with soil temperature (ST). Temperature changes may affect plant carbon–nitrogen metabolic processes; when soil temperature (ST) rises, it may induce changes in plant leaf carbon–nitrogen metabolism, thereby increasing LCNR, reflecting the influence of temperature on the carbon–nitrogen metabolic balance in leaves. Root length (RL) is positively correlated with soil temperature (ST), indicating that rising temperatures may promote root elongation. Under suitable temperatures, root cell activity increases, accelerating cell division and elongation, which facilitates root length growth; simultaneously, temperature may also affect the availability of nutrients in the soil and the root system’s nutrient uptake, thereby indirectly influencing root length. Root tissue density (RTD) is negatively correlated with soil temperature (ST), suggesting that rising temperatures may reduce root tissue density. Higher temperatures may accelerate root growth, leading to faster cell division and elongation, which results in relatively looser root tissue and lower density; they may also affect nutrient synthesis and accumulation in the root system, causing changes in root tissue density.

## 3. Discussion

### 3.1. Patterns of Variation in Functional Traits of Plant Species in Different Types of Marshes

The variation in plant functional traits reflects species’ capacity to adapt to environmental heterogeneity. Across the three marsh types, the sampled dominant herbaceous species exhibited distinct community-level patterns of leaf and root trait variation. These patterns integrate differences in dominant-species composition and trait variation within the sampled communities and may be jointly associated with environmental filtering and functional constraints [19].

Habitat-specific selective pressures led to distinct variation patterns. In inland salt marshes, leaf and root morphological traits showed the highest variability, with coefficients of variation reaching 170.32% and 113.60%, respectively. This suggests that plants under saline-alkali stress may adopt multiple adaptive strategies, potentially regulating leaf area to reduce transpiration while adjusting root structure to maintain water balance and ion uptake [20]. In forest marshes, variation was mainly concentrated in root specific length (97.43%), while leaf traits remained relatively stable, suggesting that functional variation in forest marsh communities was more strongly expressed in root traits related to resource acquisition under shaded conditions. In freshwater herbaceous marshes, both leaf area (82.60%) and root surface area (91.69%) showed high variability, which may reflect adaptations to fluctuating water levels.

Overall, functional trait variation follows a unified adaptive pattern: carbon stoichiometric traits remained comparatively stable because carbon forms the structural backbone of cell walls and supporting tissues and is constrained by conservative construction requirements and physiological homeostasis. By contrast, morphological traits such as leaf area, specific leaf area, root length, and root surface area directly determine light, water, and nutrient acquisition and can be rapidly adjusted as environmental conditions change. Their greater variability is therefore consistent with economic-spectrum theory, in which plants alter resource-acquisition structures more readily than core structural carbon investment. These findings highlight the habitat-dependent nature of trait variation and provide a theoretical basis for understanding plant adaptation strategies in marshes.

### 3.2. Trade-Offs Between Leaf and Root Traits in Wetland Plants

The theory of organ economy suggests that plants face a fundamental trade-off between resource acquisition and maintenance costs [21]. The cross-organ correlation analysis in this study (Figure 1, Figure 2 and Figure 3) suggests that although the association patterns between aboveground and belowground traits in the three types of marshes herbaceous plants appear different, they are governed by a common mechanistic framework: the organ-coordinated stress gradient regulation model. The core logic of this model is that the intensity of environmental stress determines carbon allocation priorities, which in turn regulates the mode of organ coordination [22].

The classification of stress gradients across the three types of marshes is clearly grounded in soil environmental conditions (Figure 4). Inland salt marshes exhibit significantly higher electrical conductivity than the other two wetland types, with soil C/N and C/P ratios at relatively high levels, indicating severe salinity stress and nutrient limitation; they are classified as high-stress habitats. Forest marshes have significantly higher total nitrogen, total phosphorus, and total organic carbon content in their soils compared to the other two wetland types, with abundant nutrient resources, and are classified as low-stress habitats. Soil moisture content in freshwater herbaceous marshes is significantly higher than in the other two wetland types, but temperatures are lower; nutrient levels fall between the two extremes. These habitats must simultaneously cope with waterlogging-induced anoxia and low-temperature constraints, classifying them as moderate-stress habitats. This classification aligns closely with the range of plant trait variation (Figure 5, Figure 6, Figure 7 and Figure 8) and the RDA explanatory power (Figure 9, Figure 10 and Figure 11).

In inland salt marsh habitats, high salinity stress acts on plants through both osmotic stress and ionic toxicity [23]. One possible interpretation is that plants may redirect resources toward the root system, limiting leaf area and enhancing root extension and potassium uptake, potentially as a response to osmotic stress. The lack of significant correlations between leaf and root morphological traits suggests that plants no longer pursue morphological coordination among organs but instead prioritize maximizing root stress resistance as their core objective. In the low-stress habitat of forest swamps, the correlations between aboveground and belowground traits are the strongest. Under low-stress conditions, there are no dominant stress factors, and carbon allocation pressure is low, which may allow plants to maximize growth efficiency. Sufficient photosynthetic products from the leaves support root growth, while a well-developed root system efficiently absorbs nutrients and feeds them back to the leaves, resulting in a close association between the stoichiometric characteristics of the organs. In freshwater herbaceous marshes—a moderately stressful habitat—plants cannot simultaneously maximize both hypoxia tolerance and light-competition capacity, which may force trade-offs between roots and leaves.

By placing the three wetland types on a unified environmental stress gradient, this suggests a possible continuous pattern of organ coordination: under low stress, bidirectional coordination occurs, with positive feedback between organs; under moderate stress, competition leads to a trade-off between organs; and under high stress, a unidirectional shift occurs, with resources concentrated in key stress-resistant organs. The individual differences among the three wetland types are, in essence, specific manifestations of this common mechanism across different stress gradients, addressing the question of how plants adapt to diverse habitats by adjusting the coordination between their aboveground and belowground organs.

### 3.3. Responses of Plant Functional Traits to Soil Environmental Factors in Marshes

Soil provides the fundamental basis for plant acquisition of nutrients and water, while plant functional traits represent the concrete outcomes of long-term adaptation to soil environmental heterogeneity [24]. To clarify how soil environmental factors were associated with trait variation in the three studied marsh systems, this study applied redundancy analysis (RDA).

The results showed that plants in different marshes developed adaptive strategies centered on source–sink balance through coordinated regulation of leaves and roots [25]. In inland salt marshes, increasing soil temperature promoted a higher leaf carbon-to-nitrogen ratio, suggesting a possible shift toward greater relative carbon investment and reduced nitrogen consumption. Meanwhile, root tissue density decreased with rising temperature, which may reflect a shift from structural maintenance toward root elongation, potentially as a means to avoid surface soil zones with high salt concentrations. Electrical conductivity was negatively correlated with total leaf carbon content, leaf area, and root carbon-to-nitrogen ratio, consistent with the known effects of salinity on photosynthetic carbon fixation. Soil moisture had a limited effect on leaf morphology but was positively correlated with root potassium content, raising the possibility that plants enhance root potassium uptake to alleviate salt-induced physiological drought.

In forest marshes, the leaf carbon-to-phosphorus ratio was negatively correlated with soil temperature, which may indicate accelerated phosphorus turnover under warmer conditions. Leaf area per unit mass varied with light availability and increased under better light conditions to enhance photosynthetic capacity. Root tissue density was negatively correlated with soil moisture, which may indicate reduced construction costs and expanded absorption range in humid environments. Multiple root traits were jointly affected by soil carbon, nitrogen, phosphorus, moisture, and electrical conductivity, suggesting that roots may function as multi-factor integrators shaped by both resource distribution and mechanical constraints.

In freshwater herbaceous marshes, leaf area was negatively correlated with leaf nitrogen and phosphorus contents, which may reflect a nutrient-saving strategy in relatively nutrient-poor environments [26]. The development of aeration tissues was a key mechanism for adapting to hypoxic conditions, raising the possibility that oxygen acquisition drives root architecture. Leaf traits were influenced by relatively concentrated environmental factors, whereas root traits responded to more diverse factors, implying differentiated aboveground and belowground responses.

Overall, basic resource factors, especially the soil carbon-to-nitrogen ratio, were associated with plant trait variation across the three studied marsh systems. In waterlogged wetland soils, oxygen limitation slows microbial decomposition and nutrient mineralization; consequently, soil C:N integrates organic-matter quality, nitrogen mineralization and immobilization, and the resulting availability of nitrogen to plants. Thus, soil C:N may represent an informative environmental correlate of nutrient-related trait variation, although its associations with particular traits differed among habitats. Specific stress factors may direct plant plasticity toward different functional dimensions. This pattern is consistent with the view that plants may prioritize survival over metabolism and metabolism over growth, achieving optimal allocation of carbon, nitrogen, and phosphorus through coordinated source–sink regulation between leaves and roots. An important limitation of this study is that dominant-species composition differed among the three marsh habitats, and each marsh type was represented by a single geographic area. Therefore, the observed trait patterns are interpreted at the community level for the sampled dominant herbaceous species, and the present design cannot separate species turnover from within-species trait variation. Other limitations include single-season sampling, incomplete coverage of environmental drivers, and the correlational nature of the analyses. In addition, several potentially important wetland environmental variables, including soil pH, redox conditions, inundation characteristics, ionic composition, light availability, and microtopography, were not measured in the present study. Therefore, the observed trait–environment relationships should be interpreted as associations with the measured environmental variables rather than as a complete mechanistic characterization of plant adaptation.

## 4. Materials and Methods

### 4.1. Overview of the Study Area

Northeast China is a major wetland region, spanning Heilongjiang, Jilin, and Liaoning provinces (38°42′–53°55′ N, 115°52′–135°09′ E). It contains diverse wetland types, including marshes, lakes, rivers, estuaries, tidal flats, shallow marine waters, reservoirs, ponds, and paddy fields [27]. The total wetland area is 7.5 × 104 km^2^, accounting for 14.1% of China’s wetlands and 48.3% of its marshes [28,29]. Major marshes are distributed across the Sanjiang Plain, Songnen Plain, Lower Liao River Plain, Greater and Lesser Khingan Ranges, and Changbai Mountains. The Sanjiang Plain is the largest plain marsh area in China, with about 1.12 × 10^6^ hm^2^ [30].

The western Songnen Plain, a semi-arid and ecologically fragile region, is a key inland salt marsh area, including important wetlands such as Zhalong, Momoge, and Xianghai [31]. Momoge National Nature Reserve (122°27′–124°04′ E, 45°42′25″–46°18′0″ N) covers 144,000 ha and serves as a critical stopover for migratory waterbirds [32]. Its heterogeneous habitats, formed by rivers, lakes, and depressions, are dominated by sedge wetlands, reed wetlands, and Salicornia salt marshes [33,34].

The Lesser Khingan Range extends through Heilongjiang Province and connects several major geographic units [35,36]. Heilongjiang Youhao Nature Reserve (48°13′07″–48°33′15″ N, 128°10′15″–128°33′25″ E) features gentle terrain, broad valleys, and a cold-humid climate, supporting extensive marshes, including forest, shrub, and freshwater herbaceous types, with particularly diverse forest bogs [37,38,39].

The Sanjiang Plain, formed by alluvial deposits of the Songhua, Ussuri, and Heilongjiang Rivers, covers 10.89 × 104 km^2^ and hosts China’s largest freshwater herbaceous marshes [40]. The Sanjiang National Nature Reserve, established in 1994, spans 198,089 ha and primarily protects marsh ecosystems [41].

### 4.2. Plot Setting and Sampling

**Figure 12:**
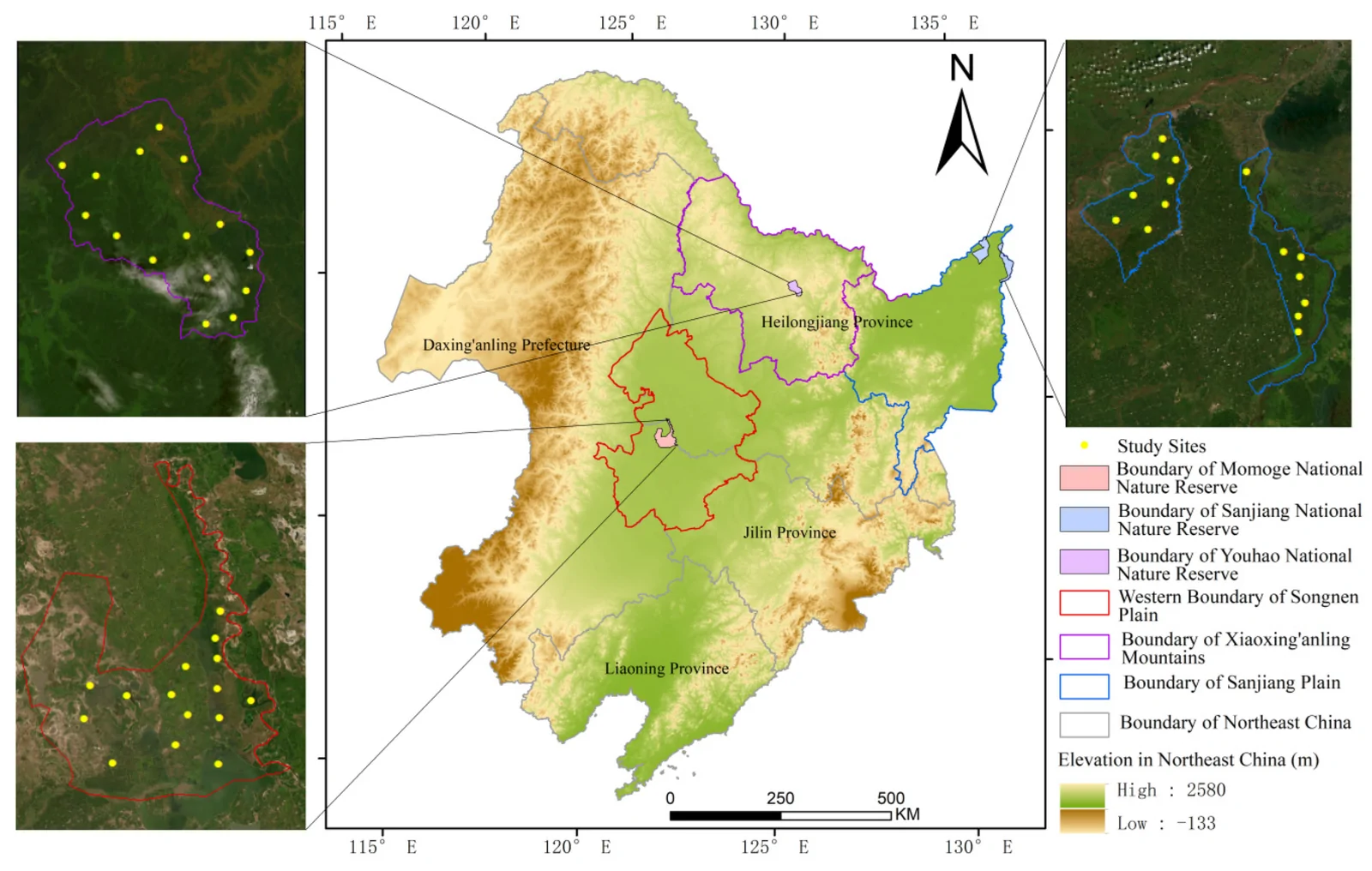
Geographic location of the study area.

Based on preliminary literature reviews and field surveys, inland salt marshes on the Songnen Plain (Momoge Nature Reserve), forest marshes in the Lesser Khingan Mountains (Heilongjiang Youhao Nature Reserve), and freshwater herbaceous marshes on the Sanjiang Plain (Sanjiang Nature Reserve) were selected as study sites (Figure 12). These areas were selected as representative marsh systems corresponding to the three targeted marsh types in Northeast China. To minimize human disturbance, plot selection criteria strictly limited sites to typical, relatively pristine habitats located far from roads and minimally affected by grazing or agricultural development. Following China’s second national wetland survey classification [42], plots were first distinguished by woody canopy cover (>30%). Herbaceous plots without canopy were classified as freshwater herbaceous marshes when EC was <200 µS/cm and pH was near neutral, and as inland salt marshes when EC was >2000 µS/cm and pH was >7.8. Plots with canopy cover >30% and EC between 200 and 2000 µS/cm were classified as forest marshes, where shading was the principal limiting factor.

The field investigation and sampling work began at the end of July 2024. According to the investigation plan formulated earlier, 45 sampling points were set up in the Momo Ge Nature Reserve, the Youyi Wetland, and the Sanjiang Nature Reserve’s riverine wetlands for the investigation. Each sampling point consisted of a 10 m by 10 m survey area, which was divided into three 1 m by 1 m sub-squares along the diagonal. Three to five dominant herbaceous plants were selected from each sampling area. The dominant herbaceous plants in the inland salt marsh area were *Phragmites australis* and *Echinochloa crus-galli* (L.) P. Beauv., etc.; in the forest marsh area, they were *Calamagrostis epigeios* and *Carex appendiculata*, etc.; and in the freshwater herbaceous marsh area, they were *Carex lasiocarpa* and *Typha orientalis* Pres, etc. These species were used to determine their functional characteristics. All statistical analyses were based on the average values of the plots. Subplots and individual plants were regarded as sub-samples used to characterize each plot and were not considered as independent observations. Soil samples were also collected, and the longitude and latitude of each sampling point as well as the soil temperature were recorded.

### 4.3. Measurement of Plant Sample Characteristics

In each plot, three to five healthy, intact individuals were selected. Root systems were excavated, and only fine roots (<2 mm) were retained, with coarse and dead roots manually removed. Samples were transported to the lab in cold storage. Leaves and fine roots were scanned using an Epson scanner; leaf images were processed with ImageJ (version 1.54f) to obtain individual leaf area (ILA), and root images with WinRHIZO (version 2021a) to obtain root length (RL), root volume (RV), and root surface area (RA). Root tissue density (RTD) was calculated as root dry mass per unit root volume (g·cm^−3^). All root measurements followed standardized fine-root trait protocols [43]. Other trait measurements are as follows:

(1) Leaf dry matter content (LDMC): Place fresh leaves in a self-sealing bag, add water to moisten them, and allow them to absorb water until saturated. After 12 h, weigh them on an electronic balance with a precision of 0.01 g; this is recorded as the fresh leaf weight. After weighing, place them in an oven at 65 °C and dry for 48 h; weigh them again on the electronic balance, and record this as the dry leaf weight. LDMC = Leaf dry weight (g)/Saturated fresh leaf weight (g)
(2) Specific leaf area (SLA): SLA = Leaf area (mm^2^)/Leaf dry weight (mg)
(3) Specific Root Length (SRL): Dry the whole roots in a 65 °C oven for 48 h, then weigh the dry weight on an electronic balance with a precision of 0.01 g. SRL = Root Length (cm)/Root Dry Weight (g)
(4) Root Tissue Density (RTD): RTD = Root Dry Weight (g)/Root Volume (cm^3^)
(5) Specific Root Area (SRA): SRA = Root Area (cm^2^)/Dry Root Weight (g)

The plant samples were washed with deionized water, blanched at 105 °C for 30 min, dried at 65 °C until constant weight was achieved, ground through a 40-mesh sieve, and stored for use. 0.2000 g of the dry sample was weighed, and then digested with H_2_SO_4_-H_2_O_2_, diluted, and filtered to prepare the test solution for the determination of total phosphorus and total potassium. Total carbon and total nitrogen were simultaneously determined using an elemental analyzer (Vario EL III, Elementar, Langenselbold, Germany), with 950 °C pure oxygen combustion and thermal conductivity detector quantification. Total phosphorus was determined using the vanadium-molybdenum yellow colorimetric method, at 440 nm; total potassium was determined using the flame atomic absorption method, at 766.5 nm (with CsCl added to inhibit ionization). For each batch of samples, a reference blank was set, the national standard substance (GBW07603) was used for concurrent calibration, parallel duplicate samples (*n* = 3) were prepared, and spiked recovery tests were conducted. The spiked recovery rate was controlled within 95% to 105%, and the parallel relative standard deviation was ≤3%. All the dete rmination results were expressed based on the dry matter mass, with the unit being g/100 g.

### 4.4. Measurement of Environmental Indicators

Within each 10 m × 10 m plot used for the plant community survey, soil samples were collected from the 0–20 cm soil layer using a soil corer. Three soil cores were collected within each plot and thoroughly homogenized to form one composite soil sample representing that plot. The same sampling depth and sampling procedure were applied consistently across all three study sites. Soil moisture (SW), soil temperature (ST), and soil electrical conductivity (EC) were measured in situ using a multi-parameter rapid soil analyzer.

After collection, soil samples were air-dried, dried at 105 °C for 24 h, ground and homogenized using a ball mill, and passed through a 100-mesh sieve for nutrient analysis. Soil organic carbon (SC) was determined using a total organic carbon analyzer, while soil total nitrogen (SN) and total phosphorus (SP) were determined using an elemental analyzer. Before each analytical batch, instrument baseline and zero-point stability were checked using blanks, and instrument responses were calibrated and verified using appropriate reference standards according to the manufacturers’ procedures. Formal sample measurements were initiated only after the reference measurements fell within the acceptable range. Blank samples, reference standards, and replicate samples were included during analysis to monitor background contamination, instrumental drift, and analytical repeatability. Samples were reanalyzed when the relative deviation between replicate measurements exceeded 5%. The same sample pretreatment, instrumental procedures, calibration, and quality-control criteria were applied to soil samples from all three study sites.

### 4.5. Data Analysis

Before analysis, trait values that did not conform to a normal distribution were log10-transformed to improve normality. To account for nested sampling, each plot was regarded as an independent observation unit; the data from subplots and individual plants within each plot were averaged to obtain the trait values at the plot level, which were not treated as independent repeated samples. The degree of variation in leaf morphology, root morphology, and chemical stoichiometric traits was calculated using the coefficient of variation (CV). The trade-off relationship between aboveground and belowground traits of plants and their correlations with environmental factors were analyzed using Pearson correlation analysis. To account for multiple testing in the correlation matrices, *p* values were adjusted using the Benjamini–Hochberg false discovery rate (FDR) correction, and statistical significance was assessed based on the adjusted *p* values (adjusted *p* < 0.05).

To compare soil environmental variables and plant functional traits among the three wetland types, the assumptions of one-way analysis of variance (ANOVA) were first examined. The normality of model residuals was assessed using the Shapiro–Wilk test, and homogeneity of variance among wetland types was evaluated using Levene’s test. When the assumptions of normality and homogeneity of variance were satisfied, one-way ANOVA was used for comparisons among wetland types. When a significant overall difference was detected, Tukey’s HSD post hoc test was further applied for pairwise comparisons with adjustment for multiple comparisons. Statistical significance was set at *p* < 0.05.

Redundancy analysis (RDA) was used separately for each marsh system to assess multivariate associations between plant functional traits and soil environmental variables. Prior to RDA, plant trait and environmental variables were standardized to eliminate differences in measurement scale. All measured soil environmental variables were retained in the RDA models to maintain consistency in environmental-variable representation among the three marsh systems. The statistical significance of the overall RDA model, individual constrained axes, and environmental variables was evaluated using permutation tests with 999 permutations. In addition to the conventional coefficient of determination (R^2^), adjusted R^2^ was calculated to provide a more conservative estimate of the proportion of trait variation explained by the environmental predictors. RDA results were interpreted as multivariate associations between environmental conditions and plant functional trait variation rather than as direct evidence of causal regulation. All data were analyzed and figures were drawn using Origin 2021, SPSS 25.0, Canoco 5, and R 4.1.3.

## 5. Conclusions

This study investigated the shaping effects of different marsh habitats on plant organ traits and analyzed the responses of plant traits to soil factors as well as their adaptive strategies. The findings hold significant theoretical implications for maintaining wetland ecosystem functions, conserving plant diversity, and regulating ecological balance. The main conclusions are as follows:

(1) The sampled dominant herbaceous communities exhibited distinct functional trait patterns among marsh habitats. In inland salt marshes, salinity and alkalinity stress caused the most dramatic morphological variations in leaves and roots; forest marshes exhibited significant plasticity in traits related to underground space exploration; and freshwater herbaceous marshes showed prominent variation in above-ground light-capturing traits. Carbon exhibited extremely strong stoichiometric stability across all organs.
(2) Wetland herbaceous communities exhibited habitat-associated cross-organ trade-off patterns in economic traits among the sampled dominant species. Plants in inland salt marshes adopt a defense strategy of reducing leaf size and concentrating resources on root development; forest marsh plants rely on a well-developed and loose root network to achieve efficient resource coupling between aboveground and belowground parts; freshwater herbaceous marsh plants tend to maximize leaf area to gain a light advantage, resulting in a pattern of relatively diluted nitrogen and phosphorus nutrients per unit area.
(3) The soil carbon-to-nitrogen ratio showed recurrent associations with plant trait variation across the three representative marsh systems studied in Northeast China, while the associated trait patterns differed among habitats. In high-stress inland salt marshes, soil C:N mainly constrains root stress-resistance traits and carbon allocation, supporting potassium uptake and osmotic adjustment under saline–alkaline conditions. In moderate-stress freshwater herbaceous marshes, it interacts with waterlogging and temperature to balance root aeration, elongation, and leaf–root carbon–nitrogen metabolism. In low-stress forest marshes, it primarily regulates nutrient supply and the coordinated growth efficiency of aboveground and belowground organs. This stress-dependent shift links the common soil driver to distinct organ-level adaptation pathways.

## Data Availability

The data generated and analyzed by this research, including the measurement data of plant leaf and root functional characteristics collected from 45 sampling points in three typical marsh areas in northeastern China, as well as soil environmental variables, can all be provided by us. These data have not yet been stored in public databases. Upon reasonable request, anonymized field-level traits and soil data can be provided by the corresponding author.

## Abbreviations

The following abbreviations are used in this manuscript:

LES	Leaf Economics Spectrum
RES	Root Economics Spectrum
